# Colonic delta-shaped anastomosis using linear staplers in laparoscopic colectomy

**DOI:** 10.1007/s10151-020-02341-3

**Published:** 2020-09-10

**Authors:** J. Y. Tajima, S. Nagayama, Y. Hiyoshi, T. Mukai, T. Nagasaki, T. Yamaguchi, T. Akiyoshi, T. Konishi, Y. Fukunaga

**Affiliations:** Department of Gastroenterological Surgery, The Cancer Institute Hospital, 3-8-31 Ariake, Koto-ku, Tokyo, 135-8550 Japan

Although laparoscopic surgery has been the standard for colon cancer as a minimally invasive procedure, intestinal anastomosis is often performed extracorporeally, because it is simple and safe. For secure extracorporeal anastomosis (EA), however, wider dissection of the intestine is required and this more extensive dissection is often labor-intensive and time-consuming, especially in obese or transverse colon cancer patients. Excessive traction of the intestine in EA can also cause damage to the intestine or unnecessary bleeding. To alleviate these difficulties, intracorporeal anastomosis (IA) is recognized as a promising option for reducing the surgical invasiveness of the procedure and for minimizing organ damage and excessive bleeding, thereby reducing short-term morbidity [1]. For colon cancers, IA is performed using either a functional end-to-end anastomosis (FEEA) or a side-to-side anastomosis (overlap) approach. On the other hand, IA in laparoscopic distal gastrectomy for gastric cancers is commonly performed using a delta-shaped anastomosis in Billroth-I reconstruction (Delta-G), as first reported by Kanaya et al. [2]. In addition, for the reconstruction of the cervical esophagus and digestive tract, an esophageal delta-shaped anastomosis (Delta-E) has been developed as a new method of stapled anastomosis [3]. With the widespread application of the delta-shaped anastomosis using linear staplers in the reconstruction of the upper digestive tract, we applied this secure and reliable approach to intestinal reconstruction following colon resection.

This video shows a colonic delta-shaped anastomosis (Delta-C) approach using linear staplers in the right and left hemicolectomy. In the right hemicolectomy, a 12-mm port for the surgeon’s right hand is placed approximately four fingerbreadths cranial to the 5-mm port placed in the left lower abdomen. Two 5-mm ports for the first assistant are placed in the right lower abdomen symmetrically opposite the two ports for the surgeon. The camera port is at the umbilicus and the surgery is performed via these five ports. The surgeon stands on the left side of the patient, the first assistant on the right, and the second assistant (camera operator) stands between the patient’s legs or on the left side depending on the situation.

After the oral and anal sides of the intestine have been transected using 60-mm linear staplers (Endo GIA™ 60-mm Articulating Medium Thick Reload with Tri-Staple™ Technology, Medtronic, Minneapolis, MN, USA), a small hole is created at the cut end of colon on the mesentery side for the insertion of a 45-mm linear stapler with the pre-attached bio-absorbable reinforcement material (Endo GIA™ 45 mm Articulating Medium Thick Reinforced Reload with Tri-Staple™ Technology, Medtronic, Minneapolis, MN, USA), which is useful for more secure stapling [4]. After the larger anvil of the linear stapler is inserted through the hole at the cut end of the colon, the smaller anvil of the stapler is inserted through the hole at the cut end of the small intestine with the help of the first assistant. Both ends are then precisely adjusted, so that the angle between the stapling line and the intestinal stump is about 45°. It is important to ensure that the stapling line is not created along the attachment of the mesentery to avoid damaging the mesentery which could cause ischemic injury. While ensuring that the cut ends do not slip down, with the cooperation of the first assistant, a linear stapling of 45 mm length between the two intestines is performed. Following the secure stapling of the two intestines, the insertion hole is at first temporarily closed by three full-thickness stitches using a single barbed suture. The key point is that the edges are staggered to prevent any overlapping of the two stapled lines. By holding up the end of the reinforcement material with the forceps of the first assistant and surgeon, the insertion hole is then closed easily and securely with one or two linear staplers. A larger diameter of anastomosis can be created by stapling the insertion hole twice. To maximize the diameter of anastomosis, the first stapling is carried out along the edge of the hole with a 30-mm linear stapler (Endo GIA™ 30-mm Articulating Medium Thick Reload with Tri-Staple™ Technology, Medtronic, Minneapolis, MN, USA) and the second stapling is performed with a 60-mm linear stapler (Endo GIA™ 60 mm Articulating Medium Thick Reload with Tri-Staple™ Technology, Medtronic, Minneapolis, MN, USA) placed at a raised angle to close the balance of the hole. There are no additional sutures needed at the anastomotic site. Since this approach is similar to an end-to-end anastomosis, the mesentery is properly aligned and positioned. After the anastomosis is completed, the full-thickness closure of the insertion hole is checked by closely inspecting the removed redundant tissue. In the left hemicolectomy, the port placement is exactly the same as in the right hemicolectomy except that the right lower and left upper ports are 12 mm and 5 mm, respectively. The surgeon stands on the right side of the patient, the first assistant on the left, and the second assistant (camera operator) on the left side of the surgeon. Also, the steps involving stapling of the two ends of the colon closely mimic those of the ileocolic anastomosis.

According to the clinical and economic assessment performed by Roy et al. [5], despite the increased cost of using multiple staplers, mechanical stapling can be considered to be a clinically and economically favorable option compared to hand-sewn suturing for establishing anastomoses in patients undergoing right colon surgery.

We have already performed the Delta-C approach on 16 patients with colon cancers. There were no short-term complications including anastomotic leakage, anastomotic stenosis, anastomotic bleeding, intra-abdominal abscesses, wound infection, bowel obstruction, or paralytic ileus.

In the Delta-C approach, intestinal reconstruction can be completed in a straightforward way after the resection of the colon. Therefore, this approach can be applied easily and safely to any type of colon resection by not only colorectal surgeons but also general surgeons.

## Electronic supplementary material

Below is the link to the electronic supplementary material.

Supplementary file1 (MP4 338650 kb)

## References

[1] van Oostendorp S, Elfrink A, Borstlap W, Schoonmade L, Sietses C, Meijerink J, Tuynman J (2017). Intracorporeal versus extracorporeal anastomosis in right hemicolectomy: a systemic review and meta-analysis. Surg Endosc.

[2] Kanaya S, Kawamura Y, Kawada H, Iwasaki H, Gomi T, Satoh S, Uyama I (2011). The delta-shaped anastomosis in laparoscopic distal gastrectomy: Analysis of the initial 100 consecutive procedures of intracorporeal gastroduodenostomy. Gastric Cancer.

[3] Okushiba S, Yo Kawarada Y, Shichinohe T, Manase H, Kitashiro S, Katoh H (2005). Esophageal delta-shaped anastomosis: a new method of stapled anastomosis for the cervical esophagus and digestive tract. Surg Today.

[4] Kimura M, Shibata Y, Mori Y (2018). Novel attempt using bioabsorbable reinforcement material on the crotch of a side-to-side anastomosis. Indian J Surg.

[5] Roy S, Ghosh S (2015). A Yoo (2015) An Assessment of the clinical and economic impact of establishing ileocolic anastomoses in right-colon resection surgeries using mechanical staplers compared to hand-sewn technique. Surg Res Pract.

